# A convenient and efficient total solid-phase synthesis of DOTA-functionalized tumor-targeting peptides for PET imaging of cancer

**DOI:** 10.1186/s13550-019-0539-0

**Published:** 2019-09-10

**Authors:** Subhani M. Okarvi, Ibrahim AlJammaz

**Affiliations:** Cyclotron and Radiopharmaceuticals Department, King Faisal Specialist Hospital and Research Centre, MBC-03, P.O. Box 3354, Riyadh, 11211 Saudi Arabia

**Keywords:** DOTA, cyclen, Bombesin, Solid-phase peptide synthesis, Biodistribution, Tumor imaging

## Abstract

**Introduction:**

An efficient and cost-effective synthesis of the metal chelating agents that couple to tumor-targeting peptides is required to enhance the process of preclinical research toward the clinical translation of molecular imaging agents. DOTA is one of the most widely used macrocyclic ligands for the development of new metal-based imaging and therapeutic agents owing to its ability to form stable and inert complexes under physiological conditions. Although solid-phase synthesis compatible DOTA-tris-(t-Bu ester) is a commercial product, it is expensive and contain chemical impurities. There is a need to explore new and cost-effective methods for the preparation of metal chelating agents, i.e., DOTA, directly on solid support to facilitate rapid, cost-effective, and high purity preparation of DOTA-linked peptides for imaging and therapy. In the present study, we describe a facile synthetic strategy of DOTA preparation and its linkage to peptides directly on solid-phase support.

**Methods:**

Bombesin (BN) peptides were functionalized with DOTA chelator prepared from cyclen precursor on solid-phase and from commercial DOTA-tris and radiolabeled with ^68^Ga. In vitro BN/GRP receptor binding affinities of the corresponding radiolabeled peptides were determined by saturation binding assays on human breast MDA-MB-231, MCF7, T47D, and PC3 prostate cancer cells. Pharmacokinetics were studied in Balb/c mice and in vivo tumor targeting in MDA-MB-231 tumor-bearing nude mice.

**Results:**

DOTA was prepared successfully from cyclen on solid-phase support, linked specifically to BN peptides and resultant DOTA-coupled peptides were radiolabeled efficiently with ^68^Ga. The binding affinities of all the six BN peptides were comparable and in the low nanomolar range. All ^68^Ga-labeled peptides showed high metabolic stability in plasma. These radiopeptides exhibited rapid pharmacokinetics in Balb/c mice with excretion mainly through the urinary system. In nude mice, MDA-MB-231 tumor uptake profiles were slightly different; the BN peptide with Ahx spacer and linked to DOTA through cyclen exhibited higher tumor uptake (2.32% ID/g at 1 h post-injection) than other radiolabeled BN peptides investigated in this study. The same leading BN peptide also displayed favorable pharmacokinetic profile in Balb/c mice. The PET images clearly visualized the MDA-MB-231 tumor.

**Conclusions:**

DOTA prepared from cyclen on solid-phase support showed comparable potency and efficiency to DOTA-tris in both in vitro and in vivo evaluation. The synthetic methodology described here allows versatile, site-specific introduction of DOTA into peptides to facilitate the development of DOTA-linked molecular imaging and therapy agents for clinical translation.

## Introduction

**Fig. 1:**
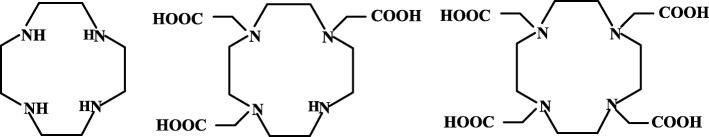
Chemical structures of (left) cyclen (1,4,7,10-tetraazacyclododecane); (middle) DO3A (1,4,7,10-tetraazacyclododecane-1,4,7-triacetic acid); (right) DOTA (1,4,7,10-tetraazacyclododecane-*N*,*N*′,*N*′′,*N*′′′-tetraacetic acid)

Timely and accurate detection of tumor lesions is a challenging task in nuclear oncology. Molecular imaging techniques, such as radionuclide imaging, have shown great clinical potential for noninvasive precise detection of tumor lesions in early stages with the use of modern and sophisticated hybrid imaging modalities, for instance PET/CT/MRI and SPECT/CT/MRI. The key requirement for any successful molecular imaging procedure is a chemical agent, called imaging probe (e.g., peptide, antibody, etc.). The macrocyclic agents capable of complexing both diagnostic and therapeutic radionuclides have shown important applications in diagnostic and therapeutic medicine in the form of metal complexes [1–4]. One of the most widely used macrocyclic ligands for the development of new metal-based imaging and therapeutic agents is DOTA (1,4,7,10-tetraazacyclododecane-*N*,*N*′,*N*′′,*N*′′′-tetraacetic acid). DOTA (Fig. 1) is an octadentate ligand with four carboxylate and four amino groups, which holds the ability to form thermodynamically stable and kinetically inert complexes with transition metals and lanthanides, under physiological conditions [5, 6].

**Fig. 2:**
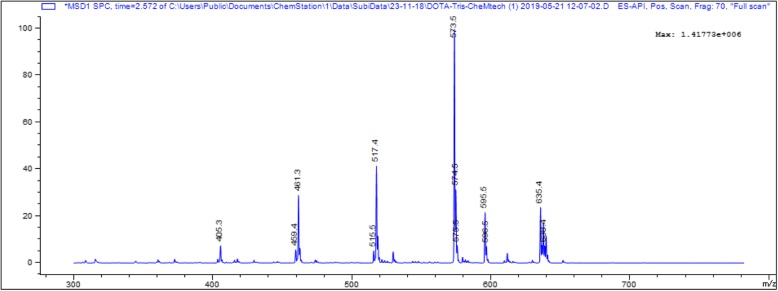
Mass spectrum profile of the commercially available DOTA-tris (t-Bu ester). As can be seen, besides the main product peak at 573, DOTA (tetraxetan) peak at 405, monoalkylating product peak at 461, and dialkylating product peaks at 517 are clearly visible in the mass spectrum. In addition, the peak found at 635 possibly correlated with tetraalkylating product 629 (M + t-Bu)^+^

Nonetheless, the commercially available macrocyclic metal chelating agents, triprotected DOTA-tris-(t-Bu ester) with a free carboxyl group forms DOTA-monoamide derivatives upon reaction with free amines [7], is one of the most frequently used bifunctional chelating agents and is fully compatible with Fmoc-based solid-phase peptide synthesis (SPPS). However, the commercial DOTA chelator is expensive and suffers from chemical impurities of both the dialkylated and tetra-alkylated cyclen [8]. An example of mass spectrometric analysis of the commercially available DOTA is presented in the Fig. 2. It can be seen that besides the main product peak at 573, a DOTA (tetraxetan) peak at 405, monoalkylating product peak at 461, and dialkylating product peaks at 517 are clearly visible in the mass spectrum. In addition, the peak found at 635 may be correlated with tetraalkylating product 629 (M + t-But)^+^. Thus, there is a need to explore alternatives route of DOTA synthesis that can provide cost-effective, high purity, and be able to attach directly to the peptide sequence during SPPS, which is the ultimate method of choice for preparing tumor targeting peptides for clinical and preclinical applications [9, 10]. Solid-phase synthesis of a wide variety of tumor-targeting peptides radiolabeled with medically useful radionuclides for diagnosis and therapy of various human diseases has opened a new scope in the field of molecular imaging [2, 5].

An effective synthetic procedure where the DOTA-linked peptide amide is synthesized on a single solid-phase support offers several advantages: the ease of coupling and time to synthesize one DOTA-peptide amide is dramatically reduced because DOTA is introduced to the peptide backbone [3, 5, 9, 10] while the peptide still attached to resin support, resulting in the formation of relatively pure DOTA-peptide product. Also, a large molar excess of DOTA will not be required as in solution-phase manual conjugation step. Owing to the well-defined peptide chemistry and excellent synthetic purity and yields obtained by the SPPS, our primary efforts in this study have been directed toward the development of DOTA-coupled peptides totally on solid-phase synthesis approach.

It is worth pointing out that direct solid-phase synthesis of DOTA-peptides is described previously [9]. Here in this study, we used bromoacetic acid instead of bromoacetyl bromide for N-terminus acylation, which is relatively easy to handle as well as provided efficient acylation and better yield, hence making this synthetic approach more suitable for preparing DOTA-coupled peptides. DOTA was efficiently prepared in the following three main synthetic steps: (i) acylation of the amino terminus of the peptide-resin with bromoacetic acid activated ester (activated in situ with HOBt/DIC). (ii) The bromoacetylated peptide-resin was then reacted with cyclen (1,4,7,10-tetraazacyclododecane) (Fig. 1) resulted in the nucleophilic displacement of bromide function with the amine of cyclen. (iii) Trialkylation of cyclen amines with tert-butyl bromoacetate followed by TFA-mediated cleavage and removal of protecting groups [11–13] (see details in “Experimental procedures” section) to afford target DOTA-linked peptides.

The main aim of this study is the comparison between the commercially available DOTA-tris-(t-Bu ester) and the one synthesized in this study on solid-phase support in order to validate and confirm that the DOTA-peptides prepared via cyclen are as potent as peptides prepared from commercially available DOTA-tris. Three new bombesin (BN) peptides analogs were selected as our model tumor targeting peptides for coupling with DOTA directly on solid phase as well as to commercially available DOTA-tris-(t-Bu ester). These BN peptides differ in terms of the presence of a specific spacer group in each peptide. It has been shown that the type and the nature of spacer group influence the receptor binding and the tumor-targeting characteristics of the resultant peptides [14, 15]. The two sets of BN peptides were then compared for their synthesis and radiolabeling characteristics, binding affinity to various BN/GRP receptor-positive cancer cell lines and in vivo biodistribution and tumor-targeting properties. The results of these comprehensive comparisons between the DOTA-peptides prepared in-house on solid-phase support and the commercially available DOTA-coupled peptides are presented in this article.

## Experimental Procedures

### General

All standard reagents, solvents, and Fmoc-amino acids for the peptide synthesis were purchased from commercial sources and used as received. DOTA-tris-(t-Bu ester) was obtained from a reputable manufacturer (Macrocyclics, Dallas, USA). ^68^Ge/^68^Ga generator was bought from Eckert & Ziegler (Berlin, Germany). The structure of each synthesized peptide was confirmed by positive-ion electrospray ionization–mass spectrometry (ESI-MS) and acquired on Agilent 6125 single quadrupole LC/MS system (Agilent Technologies, Santa Clara, CA, USA). Reversed-phase high-performance liquid chromatography (RP-HPLC) analyses were performed on a Shimadzu HPLC system (Shimadzu Corporation, Kyoto, Japan) equipped with a UV-VIS detector (Shimadzu Corporation, Kyoto, Japan), set at 220 nm, a γ radioactivity detection system, and the Lauralite chromatogram analysis program (LabLogic Systems Ltd., Sheffield, UK). Radioactive samples from in vitro and in vivo studies were measured using a γ-counter (Mucha, raytest Isotopenmessgeräte GmbH, Straubenhardt, Germany).

### Synthesis of DOTA-peptides

**Fig. 3:**
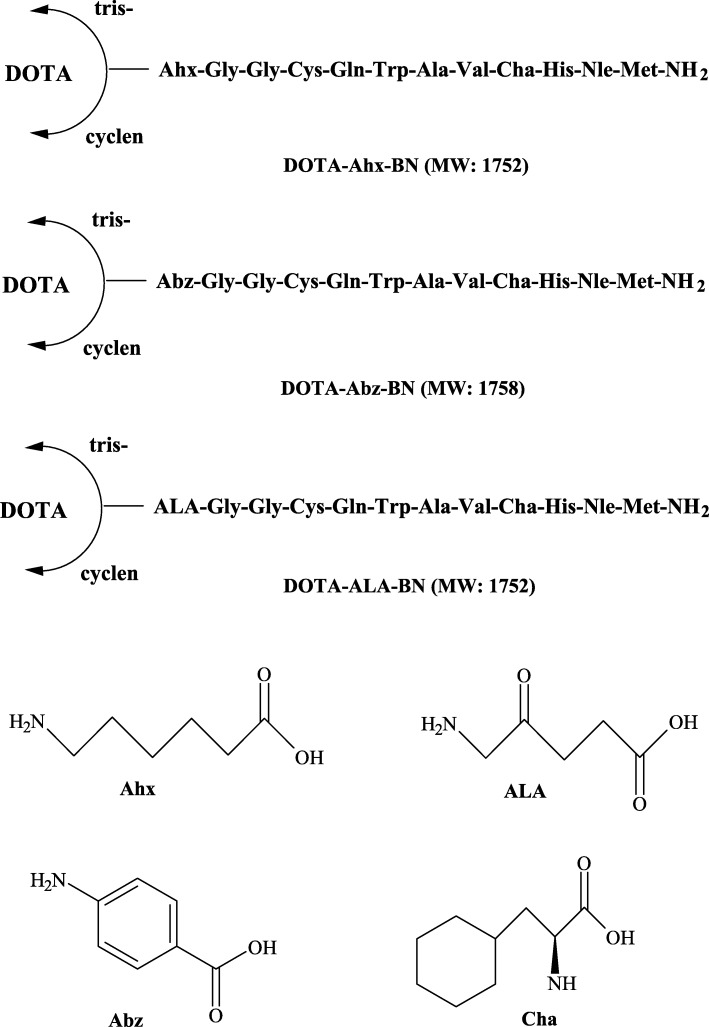
Structures of the bombesin peptide derivatives prepared by solid-phase peptide synthesis. DOTA was attached to the peptide from cyclen precursor or commercial DOTA-tris-*t*-Bu ester. *Ahx* aminohexanoic acid, *Abz* aminobenzoic acid, *ALA* aminolevulinic acid, *Cha* cyclohexylalanine

**Scheme 1:**
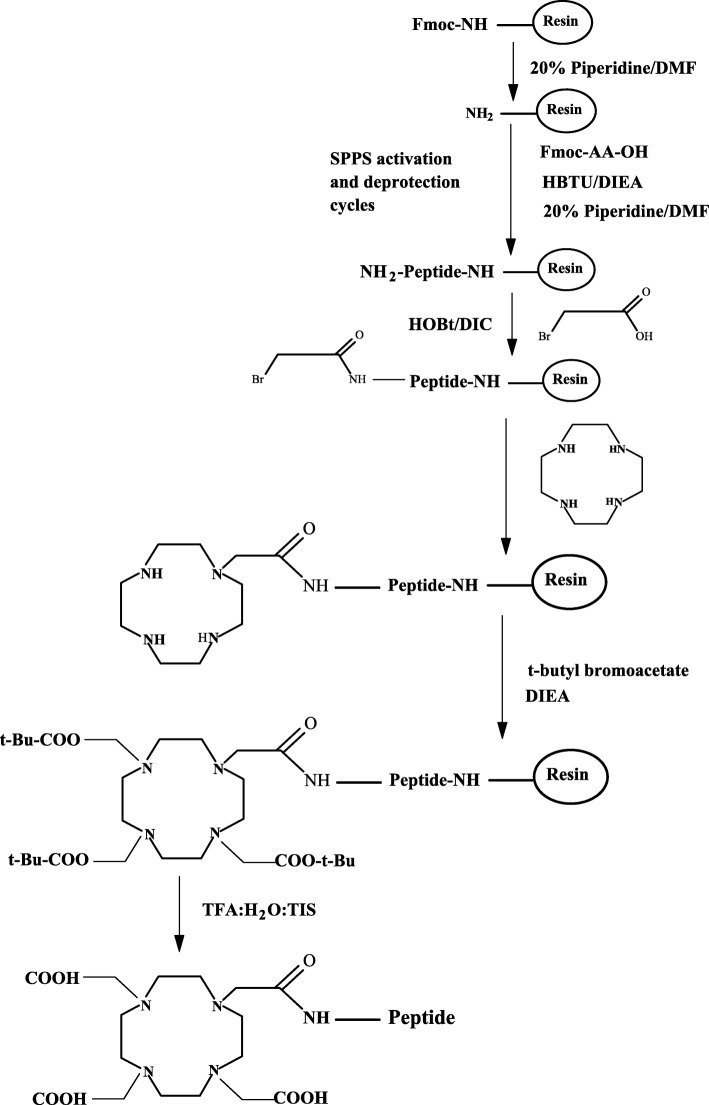
Total solid-phase synthesis of DOTA-peptides

All six BN peptides (Fig. 3) were synthesized manually using a standard peptide synthesis glass reaction vessel (Peptides International, Louisville, USA) by SPPS following standard Fmoc (9-fluorenylmethoxycarbonyl) chemistry, using Rink-amide MBHA (4-methylbenzhydrylamine) resin (100–200 mesh) on a 0.2 mmol scale according to a general method of peptide synthesis described previously [15]. Briefly, the rink amide resin (0.2 mmol, 455 mg, loading: 0.44 mmol/g) was swollen in anhydrous DMF (*N*,*N*′-dimethylformamide) for 30 min. This was followed by the removal of Fmoc-group of the resin with 20% piperidine in DMF to liberate the free primary amine from which the C-terminus of the growing BN peptide was attached. The first Fmoc-amino acid of each BN peptide sequence was activated in situ with HBTU/DIEA and mixed with the resin. The subsequent Fmoc-amino acids were sequentially coupled to the resin using the appropriate Fmoc-protected amino acids (0.8 mmol, 4 equiv.), coupling reagents HBTU (0.8 mmol, 4 equiv.), and DIEA (1.6 mmol, 8 equiv.) for 60 min. Each of the BN peptide chain was elongated in cycles of Fmoc-deprotection followed by coupling of the subsequent Fmoc-amino acid to the rink amide resin. After incorporating all the desired amino acids to the peptide sequence, the last N-terminal Fmoc-protecting group was removed to facilitate condensation of the BN peptides with DOTA ligand. The DOTA chelate was assembled in peptides during SPPS in a three-step process (Scheme 1) as described below.

#### Step 1: acylation with bromoacetic acid

The peptide resin was divided into two equal portions, each with 0.1 mmol scale. To the first portion of the peptide (0.1 mmol), the free amino terminus of the peptide-resin was first functionalized with bromoacetic acid activated ester (prepared in situ by activation of bromoacetic acid (0.5 mmol, 5 equiv. to resin) with HOBt (0.5 mmol, 5 equiv. to resin) and diisopropylcarbodiimide (DIC, 0.5 mmol, 5 equiv. to resin) in DCM/DMF (1:9 *v*/*v*, 5 mL) for 15 min prior to mixing with amine-peptide resin (pre-swell in DCM) for 90 min [13, 16, 17]. The solvent was then drained and the resin was washed with DMF and DCM. This coupling procedure was repeated once more to ensure quantitative coupling. The coupling efficiency was determined by the Kaiser ninhydrin test and a negative Kaiser ninhydrin test indicated the coupling reaction was successful. The product was used directly in the cyclen reaction step.

#### Step 2: reaction of bromoacetyl bombesin peptides with cyclen

The bromoacetylated peptide-resin (pre-swell in 5 mL DCM/DMF, 1:1 *v*/*v*) was allowed to react with cyclen (1,4,7,10-tetraazacyclododecane, 15 equiv. to the resin) (Fig. 1) dissolved in 4 mL DMF/DCM (1:1 *v*/*v*) for 3 h; resulted in the displacement of bromide function with the amine function of the cyclen [4, 9, 12]. One convenient and useful method for the synthesis of DO3A (1,4,7,10-tetraazacyclododecane-1,4,7-triacetic acid) (Fig. 1) functionalized biomolecules is the direct cyclen monoalkylation, followed by the substitution of the remaining three amine positions with *tert*-butyl bromoacetate as we have utilized this approach in the present study [4, 5, 8].

#### Step 3: trialkylation of cyclen amines with tert-butyl bromoacetate

The cyclen BN peptides were then alkylated with *tert*-butyl 2-bromoacetate (3.3 equiv. to cyclen) in the presence of DIEA (3.5 equiv. to cyclen) for 3 h, resulting in the direct alkylation of the three secondary amine positions in the cyclen ring and the formation of tert-butyl-protected DOTA-coupled peptides [3, 9, 10]. Following alkylation, the BN peptides were cleaved from the polymer support together with the deprotection of the side-chain protecting groups by the treatment with a cleavage mixture containing TFA-TIS-H_2_O (95: 2.5: 2.5) to afford the desired unprotected form of DOTA-coupled tumor-targeting peptides (Scheme 1).

The second portion of the BN peptide resin (0.1 mmol) was reacted manually with commercially available DOTA-tris-(t-Bu ester) (named here ′′tris′′). For coupling of DOTA-tris-(t-Bu ester) to free amino terminus of the peptide-resin, DOTA-tris-(t-Bu ester) (0.3 mmol, 3 equiv.) was first preactivated with HATU (0.3 mmol, 3 equiv.) in the presence of DIPEA (0.6 mmol, 6 equiv.) in DMF for 15 min prior to manual conjugation with free N*-*terminus of BN peptides bound on the polymer resin. After 5 h of coupling reaction, the coupling efficiency of DOTA to BN peptides was assessed by the Kaiser test. A negative Kaiser test indicated that the DOTA-peptide coupling was successful. Finally, the BN peptides were cleaved from the solid support with TFA-cleavage mixture as described above to provide the desired DOTA-linked peptides. The purity of each BN peptide was confirmed by HPLC analysis and their structural identity by mass spectrometry.

### Radiolabeling with ^68^Ga

For radiolabeling with ^68^Ga, a 20 μL of each DOTA-coupled BN peptide solution (1 mg/mL CH_3_CN/H_2_O (1:1)) was mixed with 400 μL of 2.5 M sodium acetate buffer, followed by the addition of ^68^GaCl_3_ (~ 5 mCi) (eluted from ^68^Ge/^68^Ga generator). The pH of the reaction mixture was adjusted between 4.5 and 5.0. The mixture was then heated for 20 min at 90 °C and cool to room temperature. The labeling mixture was filtered through a 0.2-μm pore syringe filter to remove any particulate prior to radio-HPLC analysis. ITLC-SG with 1 M NH_4_OAc:MeOH (1:1) was performed to determine colloids formation.

A nonradioactive gallium complex, ^nat^Ga-DOTA-BN was prepared by reacting for instance, DOTA-BN peptide (0.5 μM, 1 equiv.) with Ga(III)Cl_3_ (1.0 μM, 2 equiv.) dissolved in 400 μL of 0.05 M HCl. To this 400 μL of 0.2 M ammonium acetate buffer (pH 5.0) was added. Heating for 60 min at 90 °C was needed to complete the complex formation reaction.

### HPLC purification and analysis

The HPLC analysis and purification of the peptides were performed on a Shimadzu HPLC system using econosphere C18 reversed-phase column (10 μm, 250 × 4.6 mm). For all HPLC experiments, a gradient system of 0.1% (*v*/*v*) TFA in water (solvent A) and 0.1% (*v*/*v*) TFA in acetonitrile (solvent B) at a flow rate of 1.1 mL/min was used. The HPLC gradient began with a solvent composition of 95% A and 5% B from 0 to 2.5 min followed by a linear gradient of 95% A and 5% B to 5% A and 95% B over 30 min. The gradient remained at this position for 3 min before switching back to 95% A and 5% B for another 7 min. The major peak isolated and the acetonitrile was then slowly evaporated under a stream of nitrogen gas. Radiochemical purity was estimated by evaluating radioactivity peaks eluted for each ^68^Ga-labeled peptide from the HPLC column and calculating the area under the peak (ROI). The main peak of each radiopeptide was isolated and reconstituted in sterile saline for in vitro and in vivo experiments.

### Measurement of partition coefficient

For lipophilicity determination, each HPLC-purified radiopeptide (~ 20 μL, ∼ 25 μCi) was added into a glass tube containing an equal volume mixture of *n*-octanol and water (1 mL each). The samples were vigorously vortexed for 5 min and subsequently centrifuged (5000 rpm, 5 min) to yield two immiscible layers. Duplicate samples (100 μL) from each layer were carefully taken (to avoid cross-contamination between the layers) for radioactivity measurement using a γ-counter. Partition coefficient was determined by the function: partition coefficient = Log10 (radioactivity in octanol layer/radioactivity in aqueous layer) [15].

### In vitro metabolic stability in human plasma

The in vitro metabolic stability was determined by incubating each radiolabeled peptide with human plasma. The HPLC-purified ^68^Ga-radiolabeled peptides (100 μL) were incubated with human plasma (500 μL) in duplicate at 37 °C for up to 2 h. Following incubation at 1 and 2 h, the plasma proteins were precipitated with a mixture of CH_3_CN/EtOH (1:1 *v*/*v*, 400 μL) and the sample was centrifuged (7000 rpm, 7 min). The supernatant layer was removed, filtered through Millex GP filter (0.22 μm), and analyzed by radio-HPLC under the conditions described above to determine the proteolytic stability of the ^68^Ga-radiolabeled peptides.

### In vitro tumor cell binding

The cell binding of ^68^Ga-radiolabeled peptides into BN/GRP receptor-positive MDA-MB-231, MCF7, T47D human breast cancer, and PC3 human prostate cancer lines (American Type Culture Collection, Rockville, MD, USA) was performed according to the method described previously [15]. In brief, ∼ 300,000 cells (in 300 μL in low-serum media) were incubated with various amounts of radiopeptides, ranging between 0.1 and 25 nM of peptide (prepared from the serial dilutions of HPLC-purified radiopeptides), in duplicate for 60 min at room temperature. The initial concentrations of ^68^Ga-labeled peptides were determined according to a well-established HPLC technique with simultaneous detection by UV absorbance [18, 19]. As the amount of BN peptide in the reaction mixture was too small to be detected by UV absorption at 220 nm, the radioactive peptide chromatograms from the HPLC analysis were compared (with UV chromatograms) with those of known concentrations of the corresponding nonradioactive peptides for the mass determination. Incubation was terminated by dilution with cold saline (300 μL) and the cells were pelleted by centrifugation. The cell pellets was then rapidly washed with cold saline to remove any unbound peptide and centrifuged to collect supernatants. Radioactivity in the cell pellet and the washings was measured in a γ-counter. Non-specific binding was determined in the presence of ~ 200-fold molar excess of unlabeled BN peptide. Specific binding is calculated by subtracting the non-specifically bound radioactivity from that of the total binding. The dissociation constant (*K*_d_) was calculated using a plot of cell bound activity versus the concentration of the radioligand using GraphPad Prism software (GraphPad Software Inc., San Diego, CA, USA).

### In vivo animal biodistribution

Approval for the animal protocol used in this study was obtained from the Institutional Animal Care and Use Committee. Animal studies were conducted according to the international regulations governing the safe and proper use of laboratory animals [20]. In vivo biodistribution were performed on healthy Balb/c mice (*n* = 3–5 in each group, body weight 19–22 g) at 1 and 2 h after intravenous injection of the HPLC-purified radiopeptide (100 μL, 15–20 μCi, total peptide dose ~ 20 ng) via the lateral tail vein. The mice were sacrificed by cervical dislocation at specified time points and blood samples were withdrawn with a syringe from the heart. Major organs such as lungs, stomach, spleen, pancreas, intestines, liver, kidneys, etc. were isolated, weighed, and radioactivity in each organs and tissues were measured using a γ-scintillation counter. Uptake of radioactivity in the tissues and organs was expressed as the percent injected dose per gram (% ID/g) of tissue/organ, which was calculated by comparison with standard solutions representing 10% of the injected dose per animal. For the clearance studies, radioactivity in the urine with bladder and feces is expressed as the percent of the injected dose per tissue (% ID). Values are expressed as mean ± SD for a group of 3-5 animals at each time point.

### Cell lines and tumor models

BN/GRP receptor-positive MDA-MB-231 and PC3 cell lines (American Type Culture Collection, Rockville, USA) were grown as monolayers at 37 °C in a humidified atmosphere containing RPMI-1640 culture media with 10% fetal bovine serum (FBS) in tissue culture flasks. Twenty-four hours prior to conducting the tumor implantation, the media was replaced with RPMI-1640/10% FBS. The cells were grown to 80-90% confluency and harvested by trypsinization. After centrifugation, about 50 million cells were suspended in 1 mL media for implantation into mice. To prepare cells for inoculation, MDA-MB-231 or PC3 cancer cells with media were centrifuged, the media decanted, and the cell pellet was mixed with phosphate-buffered saline (PBS) to reach a concentration of approximately 5 million cells per 100 μL PBS.

Human MDA-MB-231 breast cancer xenografts female mouse models were used for in vivo tumor-targeting experiments. For the implantation of tumor xenografts, approximately five million MDA-MB-231 cells in suspension of 100 μL sterile PBS were injected subcutaneously into the left thigh of each mouse. Tumors were allowed to grow for 4-6 weeks by which tumors had reached weights of 200-300 mg. After sufficient growth of tumors, animals were injected with 20-30 μCi of the radiotracers and the animals were sacrificed at specified time points. The uptake of ^68^Ga-radiolabeled peptides in the tumors and other major body organs was calculated by γ-counting as described above.

For the receptor blocking studies, each animal was injected with excess cold of BN peptide (~ 200 μg) 20 min prior to the radiotracers injection. The animals (*n* = 2 per group) were sacrificed at 60 min post radiotracers injection and the % ID/g for the tumor and major organs was calculated as stated before.

### Nano PET/CT imaging

PET/CT scans were performed using a preclinical NanoPET/CT scanner (Mediso, Hungary) on MDA-MB-231 tumor-bearing ~ 8-week-ld female nude mice. Mice (19–23 g) were anesthetized prior to the imaging with a mixture of (100 μL, subcutaneous injection) ketamine and xylazine. One hour after the intravenous injection of radiolabeled BN peptide (100 μL peptide, 100–150 μCi, total peptide dose ~ 100 ng) via the tail vein into the anesthetized mouse, each mouse was placed in the camera in prone position and static image was acquired for 30 min. The acquired raw data in these imaging studies were reconstructed to visible image form using Interveiw Fusion software (Mediso, Hungary).

After completion of imaging, mice were sacrificed by cervical dislocation and in vivo quantitative tissue biodistribution were performed (as described above) in order to confirm and compare the findings of the imaging with quantitative tissue biodistribution.

### Statistical analysis

Results are expressed as mean ± S.D. where appropriate. For data comparisons, a Student’s *t* test was performed for comparing mean values using GraphPad Prism software (GraphPad Software, San Diego, CA, USA). A probability value (*P*) of less than 0.05 was considered statistically significant.

## Results and discussion

There is an ongoing interest in the development of new methods for facile and cost-effective synthesis of metal chelating agents, their conjugation to peptides, and ultimate use of these radiometal complexes in imaging and targeted therapy to accelerate the research and clinical translation of emerging molecular imaging agents [4, 5]. As stated earlier, DOTA is one of the most widely used macrocyclic bifunctional ligands for the development of new metal-based imaging and therapeutic agents [2, 6, 21]. DO3A-tri-t-butyl ester (1,4,7,10-tetraazacyclododecane-1,4,7-tris (t-butyl acetate)) is an important starting material for the preparation of macrocyclic chelating agents and magnetic resonance imaging contrast agents [5, 8]. Commercially available DOTA-tris-(t-Bu ester) is not an optimal ligand as it contains impurities and is costly [8]. In order to make DOTA-coupled compounds more readily available, we describe herein a simple and cost-efficient three-step solid-phase synthetic route of DOTA-linked peptide preparation starting from the condensation reaction of bromoacetylated peptides with cyclen followed by the alkylation of cyclen-peptide with ter-butylbromoacetate. We chose BN peptides as our model tumor-targeting peptides because of the overexpression of BN/gastrin-releasing peptide (BN/GRP) receptors on various human cancer cell lines including breast, prostate, and ovarian carcinomas. The preclinical evaluation and comparison of the newly synthesized DOTA-BN peptide analogs prepared from cyclen precursor and commercial DOTA-tris-(t-Bu ester) are presented in this paper.

### Synthesis of peptides

Our synthetic findings suggest that DOTA can be conveniently and successfully prepared by solid-phase synthetic method and attached to the amine terminus of the peptide-resin in satisfactory yields (up to 40%). It should be noted that for acylation reaction, chloroacetic acid can also be used efficiently and it is as potent acylating agent as bromoacetic acid [11]. Cyclen is the key precursor and provides a useful bifunctional ligand framework for synthesis of DOTA-related macrocyclic ligands. The nitrogens of the cyclen ring can be selectively alkylated with various ligating pendent groups, allowing an effective strategy for ligand design. It is worth highlighting that monoalkylation of cyclen is the crucial step during solid-phase DOTA synthesis and the selective monoalkylation of cyclen was achieved when an excess molar amount of cyclen (10-15 equiv. to resin) was used. For the cyclen alkylation step, it is imperative to use proper molar ratios of *tert*-butyl 2-bromoacetate to the resin (3.0-3.3 equiv. to resin) and optimized reaction time (~ 3 h) in order to obtain a fully alkylated target compound [9, 10, 21].

**Fig. 4:**
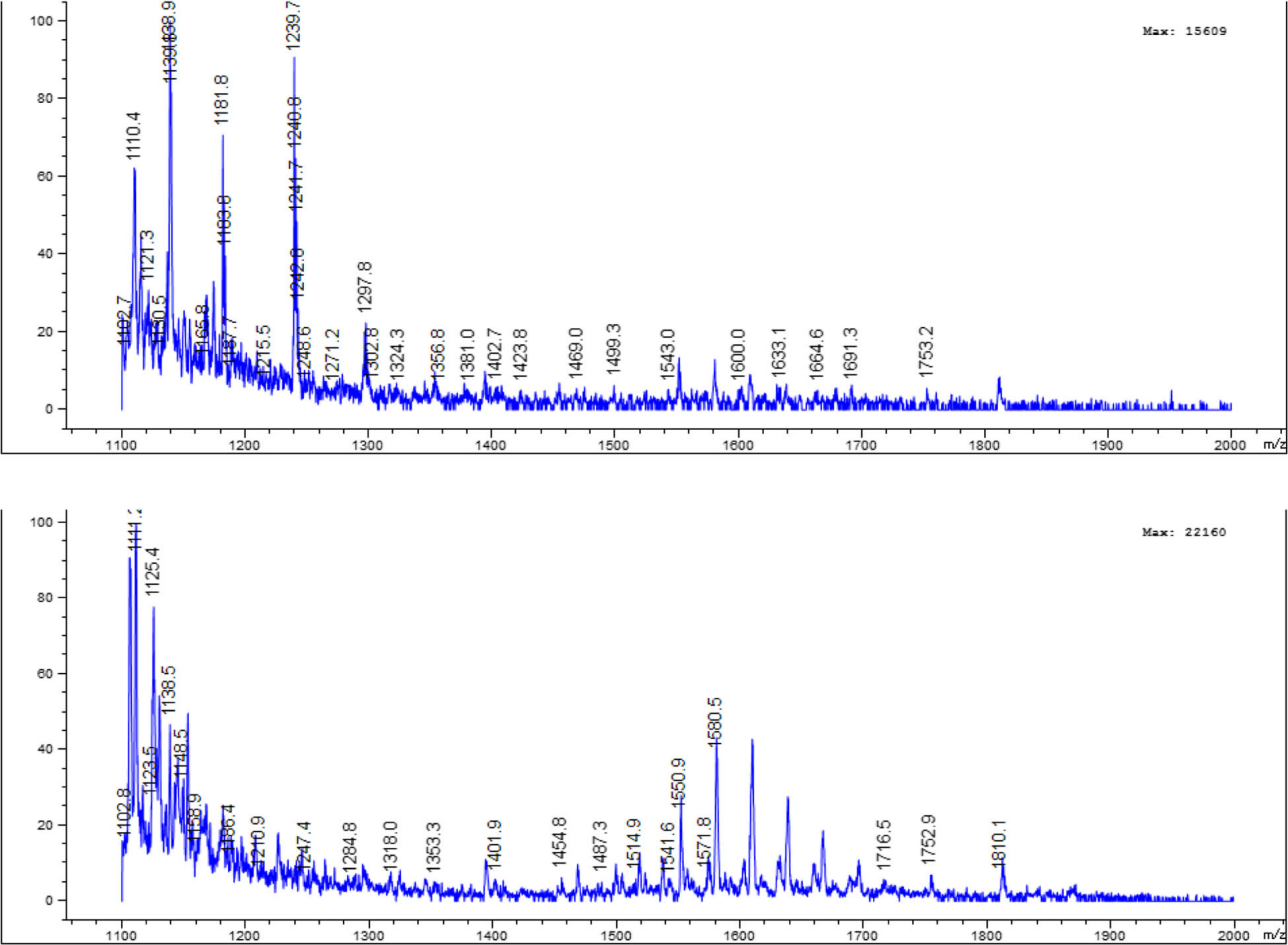
An example of the mass spectrum profile of the DOTA-Ahx-BN prepared from cyclen procedure (above) and with DOTA-tris (t-Bu ester) (below). It can be seen that no overalkylating product was observed when the DOTA-Ahx-BN synthesized by the cyclen approach. While tetraalkylating product 1813 (M + t-Bu + H)^+^ is clearly visible in the spectrum when the DOTA-peptide was prepared using DOTA-tris (t-Bu ester)

It is should be noted that when DOTA-coupled peptides are prepared from the commercially available DOTA-tris, beside the main peptide product peak, a peak at [M + 56]^+^ was usually found, indicating tetraalkylating product formation. Whereas in the case of DOTA-coupled peptides synthesized with the cyclen procedure, we occasionally see this overalkylating/tetraalkylating product. An example of the mass spectrometric analysis of DOTA-coupled peptides prepared from DOTA-tris and DOTA-cyclen is presented in the Fig. 4. The identity and purity of each peptide was confirmed by mass spectrometry and HPLC analysis. It was found that the experimentally determined molecular weights correlated well with the theoretically calculated values (Table 1).

**Table 1:** Characteristics of the ^68^Ga-labeled BN peptides

Parameter	^68^Ga-DOTA-Ahx-BN		^68^Ga-DOTA-Abz-BN		^68^Ga-DOTA-ALA-BN	
	Cyclen	Tris-	Cyclen	Tris-	Cyclen	Tris-
Labeling efficiency (%)	> 92.0	> 92.0	> 90.0	> 91.0	> 94.0	> 95.0
Radio-HPLC *t*R (min)	18.3	18.3	19.2	19.3	20.3	20.3
ESI-MS [M + H]^+^	1753.0	1753.0	1759.0	1759.0	1753.1	1753.0
Log *P* (octanol/water)	− 1.21 ± 0.11	− 1.18 ± 0.06	− 1.10 ± 0.05	− 1.04 ± 0.10	− 0.80 ± 0.05	− 0.88 ± 0.07

### Radiolabeling with ^68^Ga

**Fig. 5:**
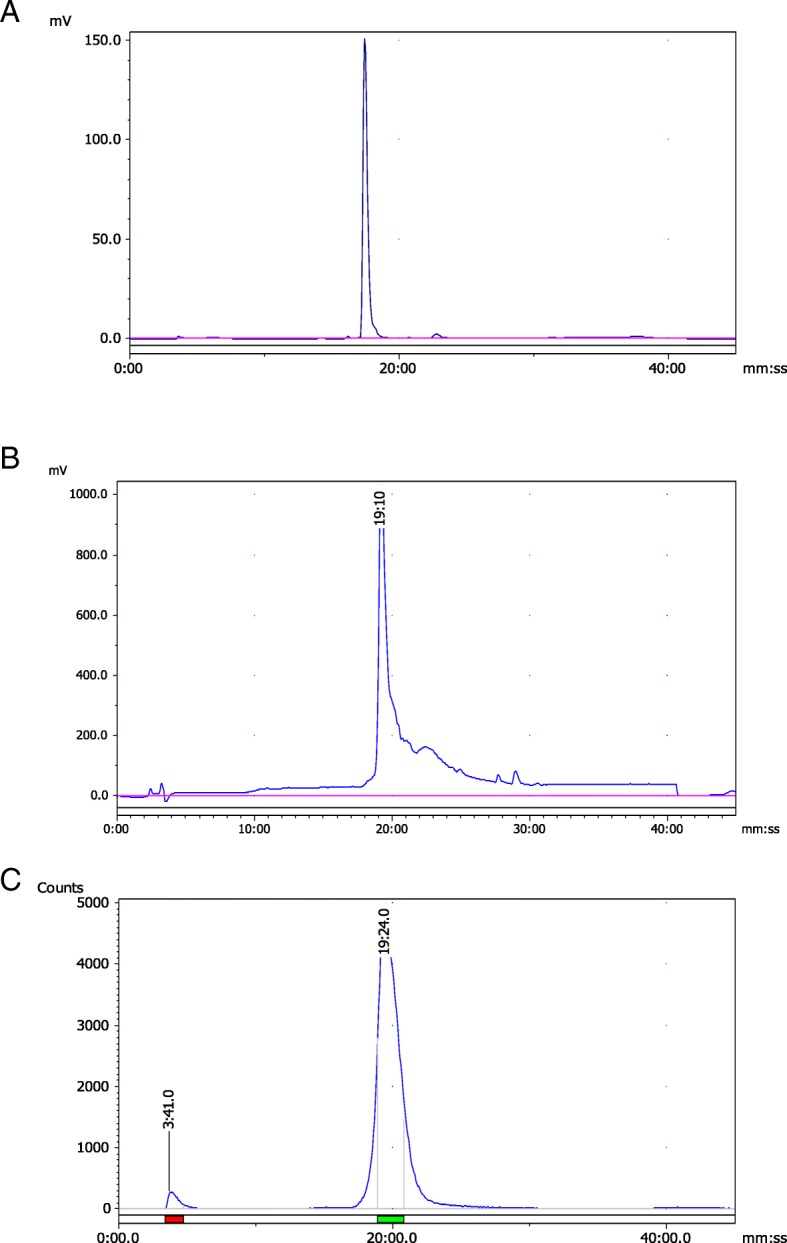
Representative HPLC chromatograms of DOTA-Ahx-BN peptide. **a** DOTA-Ahx-BN peptide (UV: 220 nm); **b** DOTA-Ahx-BN peptide after labeling with cold ^nat^Ga for identity confirmation (UV: 220 nm); **c** DOTA-Ahx-BN peptide after radiolabeling with radioactive ^68^Ga. Note: UV detector connected before radioactive detector

Radiolabeling of DOTA-coupled BN peptides with ^68^Ga was accomplished simply by mixing ~ 20 μg BN peptide with sodium acetate buffer and ^68^GaCl_3_ followed by heating to complete the labeling reaction. By this facile method, a high and reproducible radiolabeling of peptides with ^68^Ga was achieved (up to 95%), with a specific radioactivity of greater than 250 Ci/mmol. Radio-HPLC analysis profiles of each ^68^Ga-labeled BN peptides showed the formation of one major radioactive product along with some minor peaks (Fig. 5). A radiolabeling efficiency of greater than 90% was obtained in all cases, and it ranged between 90 and 95%. All the complexes were found to be stable at least 4 h after the radiolabeling as determined by radio-HPLC. RP-HPLC retention times of the major peaks, ranging from 18.3 to 20.3 min, reflected slightly variable lipophilicity of these ^68^Ga-complexes (Table 1). The colloids formation was below 2% as revealed by ITLC-SG analysis.

In addition, identification of ^68^Ga-DOTA-BN complex was achieved by comparison of the retention time with the cold ^nat^Ga-DOTA-BN compound eluted with identical retention time under the same HPLC condition. Representative HPLC analysis chromatograms are given in Fig. 5.

### Partition coefficient determination

The partition coefficient values of the six radiopeptides investigated are listed in Table 1. The lipophilicity was determined by measuring the radioactivity distribution ratios of ^68^Ga-labeled BN peptides between *n*-octanol and water layers. A small variation in the partition coefficient values was obtained for the BN peptides under investigation possibly due to the similarity of the amino acids sequence and differ mainly in terms of the specific spacer group (i.e., Ahx, Abz, and ALA) in the sequence (Fig. 3), which might contribute to slight different degrees of lipophilicity of these peptides.

The BN peptide, with Ahx spacer group was found to be the most hydrophilic (− 1.21 ± 0.11), whereas the peptide containing ALA group was less hydrophilic (− 0.80 ± 0.05) among the tested BN peptides. The obtained log *P* values varied between − 0.80 ± 0.05 and − 1.21 ± 0.11 (see Table 1), demonstrating variable lipophilicity of the ^68^Ga-complexes. In most cases, a good correlation was observed between the obtained log *P* values and the RP-HPLC retention times of the radiopeptides; as the most hydrophilic BN peptide (with Ahx group) showed the shortest retention time (18.3 min), whereas a less hydrophilic BN peptide (with ALA group) displayed a somewhat higher retention time (20.3 min) on RP-HPLC (Table 1).

The partition coefficient measurement is important criteria as it usually predicts the possible route of excretion of a radiopeptide. Peptides with high hydrophilicity are usually excreted by the urinary system, the preferred route of excretion of a tumor-targeting agent. Indeed, our biodistribution findings also demonstrated that ^68^Ga-DOTA-Ahx-BN exhibited a high degree of renal excretion, possibly due to the hydrophilic nature of the complex (Table 3).

### In vitro metabolic stability in human plasma

In order to produce maximum tumor-targeting effect, it is important that tumor-targeting peptides exhibit high metabolic stability in human plasma and reach intended target intact to deliver maximum tumor-targeting effect. The proteolytic degradation of all six radiolabeled BN peptides were investigated in vitro in human plasma. The radio-HPLC analysis revealed that more than 80% of radioactivity remained bound to peptides after 2 h of incubation, with a low reformation to free ^68^Ga and some unknown hydrophilic metabolites (up to 20%), demonstrating the high metabolic stability of ^68^Ga-labeled BN peptides. The estimated percent of radiopeptides remaining intact in plasma was found to be between 89 and 94% at 1 h and between 81 and 87% at 2 h, indicating a low enzymatic degradation of ^68^Ga-complexes by plasma proteases.

### In vitro tumor cell binding

In vitro cell binding of ^68^Ga-labeled BN peptides were tested for their ability to bind with BN/GRP receptor-expressing human breast and prostate cancer cell lines. These include estrogen receptor-positive MCF7, estrogen receptor-independent MDA-MB-231, human ductal breast carcinoma T47D, and androgen receptor-independent PC3 prostate cancer cell lines. These human cancer cell lines presented variable receptor expression for BN peptides [22, 23]. The binding of radiolabeled BN peptides to the respective receptor-positive cell lines was determined by saturation binding assays, and the results of the binding affinity (*K*_d_) and the number of binding sites/cell (*B*_max_) was determined by GraphPad software and are summarized in Table 2.

**Table 2:** In vitro tumor cell binding characteristics: determination of dissociation constants (*K*_d_) in nM and apparent number of binding sites per cell (*B*_max_) of the ^68^Ga-labeled BN peptides (*n* = 3-5 experiments; mean values ± SD; *B*_max_ SD < 15%)

		^68^Ga-DOTA-Ahx-BN		^68^Ga-DOTA-Abz-BN		^68^Ga-DOTA-ALA-BN	
		Cyclen	Tris-	Cyclen	Tris-	Cyclen	Tris-
MDA	*K*_d_	12.73 ± 2.94	11.60 ± 3.05	11.81 ± 2.11	13.10 ± 1.29	15.14 ± 3.12	14.68 ± 2.24
	*B*_max_	5.13 × 10^5^	5.27 × 10^5^	6.09 × 10^5^	5.89 × 10^5^	1.02 × 10^5^	1.12 × 10^5^
MCF7	*K*_d_	9.80 ± 1.64	9.32 ± 1.50	10.75 ± 1.85	10.98 ± 1.10	13.69 ± 2.32	12.30 ± 1.94
	*B*_max_	6.90 × 10^5^	6.22 × 10^5^	5.12 × 10^5^	4.86 × 10^5^	2.11 × 10^5^	1.05 × 10^5^
T47D	*K*_d_	15.28 ± 3.42	14.51 ± 2.40	15.04 ± 3.24	13.82 ± 2.66	15.56 ± 3.47	16.0 ± 4.01
	*B*_max_	4.75 × 10^5^	4.91 × 10^5^	1.17 × 10^5^	1.0 × 10^5^	9.90 × 10^4^	9.68 × 10^4^
PC3	*K*_d_	9.89 ± 1.56	10.45 ± 1.71	12.52 ± 2.09	11.24 ± 2.17	9.70 ± 2.01	13.49 ± 2.53
	*B*_max_	5.29 × 10^5^	4.88 × 10^5^	4.30 × 10^5^	5.90 × 10^5^	2.12 × 10^5^	2.63 × 10^5^

It is worth mentioning here that all the six BN peptides share nearly the same receptor binding sequence (Fig. 3), but differ in terms of methods of DOTA synthesis (cyclen vs. tris) and the particular spacer group introduced between the peptide sequence and DOTA chelating moiety. The results suggest that the ^68^Ga-labeled BN peptide derivatives exhibited high affinity binding (*K*_d_ values up to 16 nM) to both human breast and prostate cancer cell lines under investigation. The data demonstrate that the BN peptides based on either DOTA-cyclen and their counterparts DOTA-tris displayed quite similar binding affinities to both estrogen receptor-positive and estrogen-independent breast cancer cells, with the binding values ranging between 9.32 and 15.14 nM. A general trend of slightly lower binding affinity was observed (range, 13.82 to 16.0 nM) for all the radioconjugates toward human ductal T47D breast cancer than MDA-MB-231 and MCF7 cancer cells. In addition to good binding to breast cancer cells, the radiolabeled BN peptides under investigation showed equally good binding affinities toward androgen-independent PC3 prostate cancer cell line, with binding affinities varied from 9.70 to 13.49 nM.

The receptor density (*B*_max_) is generally found to be high to moderate for MCF7, MDA-MB-231, and PC3 and slightly low for T47D cell line (Table 2). The maximum *B*_max_ value of 6.90 × 10^5^ sites/cell was obtained for MCF7, and the lowest value of (9.68 × 10^4^ sites/cell) was obtained for T47D among the tested cell lines.

From these studies, it seems that the cell binding characteristics are not compromised by the method used for preparation of DOTA (cyclen or tris). Furthermore, despite the modifications in the peptide sequence, such as the introduction of a chelating moiety for radiolabeling and the addition of specific amino acids and spacer groups (Fig. 3), the BN peptides maintained their potency and held high affinity and specificity toward breast and prostate cancer cells. This underlines the potential of ^68^Ga-labeled BN peptides for targeting various GRP-receptor-positive cancers.

### Biological evaluation

#### Pharmacokinetic studies in normal Balb/c mice

In vivo biodistribution findings of all six ^68^Ga-labeled BN peptides in normal Balb/c mice at 1 and 2 h post intravenous injection are summarized in Table 3. In vivo biodistribution kinetics of ^68^Ga-labeled BN peptides were performed first in normal Balb/c mice (without tumors) to determine normal tissue uptake patterns and clearance kinetics. All the six radiolabeled BN peptides displayed a somewhat identical biological behavior, characterized by rapid and efficient clearance from the blood both at 1 h and 2 h post-injection (p.i.), with less than 1% ID/g remaining in the blood after 2 h p.i. The DOTA-coupled BN peptides with different degree of lipophilicity (Table 3) displayed variable uptake in the liver. The radioactivity retained by the liver ranged from 0.61 ± 0.10% to 2.30 ± 0.37% ID/g at 1 h and from 0.46 ± 0.07% to 1.75 ± 0.26% ID/g after 2 h of radiopeptide injection. Kidney was the only organ that showed the highest radioactivity accumulation, which ranged from 1.98 ± 0.34% to 5.89 ± 1.0% ID/g at 1 h and from 1.41 ± 0.27% to 3.05 ± 0.41% ID/g at 2 h p.i, demonstrating varying degree of accumulation and retention of these complexes by the kidneys (Table 3). A low to moderate uptake of radioactivity was observed in the BN/GRP receptor-expressing organ pancreas at both the time points (range, 0.82% to 2.0% ID/g). Another BN/GRP receptor-expressing organ stomach also showed relatively low accumulation of radioactivity at both the time points (up to 1.82 ± 0.36% ID/g). The accumulation in the lungs was also low (below 1.5% ID/g) at both 1 h and 2 h p.i., indicating low colloidal particles formation by these complexes. Radioactivity found in the intestines (measured without their contents), varied between 0.64 ± 0.12% and 0.88 ± 0.15% ID/g at 1 h and between 0.41 ± 0.05% and 0.73 ± 0.12% ID/g at 2 h p.i. All the radioconjugates displayed low bone activity both at 1 h and 2 h p.i. of ^68^Ga-DOTA BN peptides indicating the high in vivo stability of these complexes, which correlates well with the high metabolic stability attained in human plasma in vitro.

**Table 3:** In vivo biodistribution of ^68^Ga-DOTA-labeled BN peptides in Balb/c mice at 1 h and 2 h post-injection (*n* = 3-5). Data are expressed as % injected dose per gram of tissue (mean values ± SD). Urinary excretion values (% injected dose) were calculated by measuring the radioactivity associated with the excreted urine and bladder contents at the time of sacrifice at 1 and 2 h post-injection

		^68^Ga-DOTA-Ahx-BN		^68^Ga-DOTA-Abz-BN		^68^Ga-DOTA-ALA-BN	
	Time/p.i.	Cyclen	Tris-	Cyclen	Tris-	Cyclen	Tris-
%ID/g in:							
Blood	1 h	0.18 ± 0.04	0.29 ± 0.05	0.40 ± 0.11	0.66 ± 0.13	0.80 ± 0.16	0.88 ± 0.14
	2 h	0.11 ± 0.03	0.17 ± 0.02	0.12 ± 0.02	0.31 ± 0.05	0.51 ± 0.12	0.27 ± 0.02
Lungs	1 h	0.19 ± 0.04	0.36 ± 0.10	0.21 ± 0.05	0.58 ± 0.10	1.39 ± 0.30	0.42 ± 0.10
	2 h	0.10 ± 0.02	0.22 ± 0.05	0.13 ± 0.03	0.40 ± 0.08	0.98 ± 0.23	0.19 ± 0.03
Stomach	1 h	1.48 ± 0.22	1.71 ± 0.19	1.69 ± 0.24	1.82 ± 0.36	1.50 ± 0.37	1.44 ± 0.26
	2 h	0.43 ± 0.11	0.48 ± 0.06	0.45 ± 0.10	0.52 ± 0.12	0.44 ± 0.10	0.53 ± 0.09
Spleen	1 h	0.13 ± 0.01	0.09 ± 0.01	0.12 ± 0.02	0.11 ± 0.01	0.10 ± 0.01	0.14 ± 0.03
	2 h	0.09 ± 0.01	0.06 ± 0.0	0.07 ± 0.01	0.10 ± 0.01	0.08 ± 0.0	0.10 ± 0.02
Pancreas	1 h	1.49 ± 0.12	2.0 ± 0.27	1.07 ± 0.13	1.48 ± 0.12	1.54 ± 0.31	0.98 ± 0.13
	2 h	1.02 ± 0.15	1.25 ± 0.13	0.88 ± 0.11	0.97 ± 0.05	0.89 ± 0.12	0.82 ± 0.12
Liver	1 h	0.61 ± 0.10	1.12 ± 0.30	0.68 ± 0.16	1.59 ± 0.23	2.30 ± 0.37	1.78 ± 0.26
	2 h	0.46 ± 0.07	0.53 ± 0.09	0.50 ± 0.07	0.91 ± 0.15	1.75 ± 0.26	0.86 ± 0.17
Intestines^a^	1 h	0.64 ± 0.12	0.67 ± 0.12	0.85 ± 0.17	0.79 ± 0.16	0.88 ± 0.15	0.85 ± 0.13
	2 h	0.41 ± 0.05	0.43 ± 0.09	0.53 ± 0.14	0.61 ± 0.09	0.73 ± 0.12	0.64 ± 0.14
Bone	1 h	0.15 ± 0.03	0.17 ± 0.03	0.13 ± 0.04	0.22 ± 0.05	0.24 ± 0.04	0.14 ± 0.03
	2 h	0.06 ± 0.0	0.09 ± 0.01	0.09 ± 0.01	0.12 ± 0.01	0.15 ± 0.02	0.08 ± 0.01
Kidneys	1 h	1.98 ± 0.34	2.89 ± 0.41	2.96±0.30	4.0 ± 0.82	5.89 ± 1.0	3.83 ± 0.67
	2 h	1.41 ± 0.27	1.63 ± 0.24	1.88 ± 0.25	2.86 ± 0.37	3.05 ± 0.41	2.76 ± 0.49
%ID excreted in:							
Urine	1 h	80.0 ± 9.80	74.0 ± 8.03	70.0 ± 8.60	43.0 ± 8.70	53.0 ± 6.80	71.0 ± 12.45
	2 h	64.0 ± 6.35	69.0 ± 5.15	62.0 ± 9.54	50.0 ± 3.45	46.0 ± 9.12	78.0 ± 15.0
Feces	1 h	0.85 ± 0.17	0.83 ± 0.19	1.01 ± 0.20	0.90 ± 0.10	1.07 ± 0.17	1.03 ± 0.12
	2 h	0.76 ± 0.11	0.69 ± 0.10	0.88 ± 0.12	0.59 ± 0.08	0.91 ± 0.14	0.77 ± 0.10

^a^Parts of the intestines were measured without their contents

It is important to mention here that precautions were taken for the maximal collection of urine, but due to the practical difficulties, complete collection of urine from mice after sacrifice is not always possible, which may result in underestimation of the urinary excretion. Urinary excretion values were calculated using the radioactivity associated with the excreted urine and bladder contents at the time of sacrifice. Any urine expelled at the time of sacrifice was also collected and counted together with the excreted urine. Rapid urinary excretion of radioactivity was seen for all the radiocomplexes, with more than 40% ID already in the urine after 1 h p.i. The most favorable clearance pattern was observed for the hydrophilic (log *P* = − 1.21) BN peptide analog, with Ahx (aminohexanoic acid) spacer group, as 80% ID already in the urine at 1 h p.i. Another hydrophilic BN peptide (log *P* = − 1.10), containing Abz (aminobenzoic acid) spacer and with glycine residue adjacent to it; formulate a hippurate-like structural spacer group, exhibited equally good renal excretion properties since 70% ID was found in the urine after 1 h of radiotracer injection. A hippurate-type biomolecule is known to facilitate clearance through kidneys as is generally preferred for radiopeptides. The clearance kinetics studies highlight the significance of the degree of lipophilicity as one of the deciding factors in the clearance route of a radiopharmaceutical.

In general, a rapid and efficient clearance of radioactivity was achieved from most of the tissues/organs, highlighting the favorable biodistribution profiles of these radiolabeled BN peptides. More importantly, no major difference between the biological behavior of the BN peptides linked with DOTA from cyclen precursor and the commercial DOTA-tris-(t-Bu ester) was observed (Table 3), indicating the equal efficiency and potency of these peptides and the low impact of the method of DOTA preparation on the biological properties of the BN peptides under investigation.

#### In vivo tumor targeting

^68^Ga-labeled BN peptides were further investigated in nude mice carrying estrogen-independent and highly aggressive MDA-MB-231 xenografts in order to determine their potential for diagnosis of BN/GRP receptor-positive breast cancer in vivo (Table 4). In nude mice, all six radiolabeled BN peptides exhibited rapid and efficient clearance from the blood (< 1.0% ID/g) at both 1 h and 2 h p.i., as it was the case in the normal Balb/c mice. The highest uptake in the tumors was achieved for ^68^Ga-DOTA(cyclen)-Ahx-BN (2.32 ± 0.25% ID/g) as early as 1 h p.i, which reduced to 1.65 ± 0.19% ID/g at 2 h p.i. (with 28% washout from the tumors over 2 h). The same leading BN peptide also exhibited favorable pharmacokinetics in normal mice (Table 3). When a blocking dose of 300 μg of nonradioactive BN peptide was injected intramuscularly for in vivo saturation of receptors 20 min prior to the administration of radiopeptide, it was observed that the uptake in the tumors was reduced by approximately 80% (0.47 ± 0.06% ID/g blocked vs. 2.32 ± 0.25% ID/g unblocked, *P* = 0.002), highlighting the specificity of the ^68^Ga-labeled BN peptide for respective BN/GRP receptor-positive breast cancer cells. The corresponding ^68^Ga-DOTA(tris)-Ahx-BN also showed nearly identical tumor uptake value 2.09 ± 0.33% ID/g at 1 h and 1.62 ± 0.18% ID/g at 2 h p.i. The tumor uptake values of other BN peptides varied from 1.78 ± 0.40% to 2.15 ± 0.33% ID/g at 1 h p.i and from 0.84 ± 0.13% to 1.37 ± 0.25% ID/g at 2 h p.i. (Table 4). With the blocking dose, good reduction in the tumors (from 51 to 80%) was attained validating the in vivo tumor receptor specificity of these radiolabeled BN peptides for BN/GRP receptor-positive breast cancer cells. The accumulation in the kidneys was moderate (up to 6.52% ID/g) but still somewhat higher than the values observed in the Balb/c mice. A low to moderate uptake of radioactivity was found in the BN/GRP receptor-expressing organ pancreas (up to 2.94% ID/g) (range, 0.71 to 2.94% ID/g). It was found that the same blocking dose significantly reduced the uptake in the receptor-positive organ, pancreas, with the maximum blockade of 80% occurred for ^68^Ga-DOTA-Ahx-BN analog. Moreover, a variable influence of the blocking dose was seen in the stomach, intestines, and kidneys probably related to the low receptor expression by some of these organs [24].

**Table 4:** In vivo biodistribution of ^68^Ga-labeled DOTA-BN peptides in nude mice with MDA-MB-231 tumors at 1 h and 2 h post-injection (*n* = 3-5). Data are expressed as % injected dose per gram of tissue (mean values ± SD). The radioactivity in the urine + bladder is expressed as % injected dose per tissue

		^68^Ga-DOTA-Ahx-BN		^68^Ga-DOTA-Abz-BN		^68^Ga-DOTA-ALA-BN	
	Time/p.i.	Cyclen	Tris-	Cyclen	Tris-	Cyclen	Tris-
Blood	1 h	0.92 ± 0.15	0.70 ± 0.12	1.0 ± 0.16	0.93 ± 0.22	0.79 ± 0.13	0.85 ± 0.15
	2 h	0.51 ± 0.07	0.60 ± 0.09	0.55 ± 0.10	0.64 ± 0.16	0.37 ± 0.10	0.30 ± 0.04
Lungs	1 h	0.55 ± 0.10	1.86 ± 0.14	2.80 ± 0.55	1.82 ± 0.30	1.05 ± 0.17	2.35 ± 0.33
	2 h	0.36 ± 0.03	0.61 ± 0.07	1.15 ± 0.23	1.26 ± 0.25	0.67 ± 0.13	0.70 ± 0.16
Stomach	1 h	0.73 ± 0.13	0.87 ± 0.11	1.34 ± 0.29	1.0 ± 0.20	0.87 ± 0.19	0.83 ± 0.21
	2 h	0.34 ± 0.05	0.60 ± 0.07	0.49 ± 0.12	0.52 ± 0.13	0.46 ± 0.05	0.37 ± 0.07
Spleen	1 h	0.25 ± 0.02	0.33 ± 0.02	0.22 ± 0.07	0.18 ± 0.03	0.26 ± 0.03	0.44 ± 0.06
	2 h	0.14 ± 0.01	0.16 ± 0.01	0.12 ± 0.02	0.09 ± 0.02	0.17 ± 0.02	0.35 ± 0.03
Pancreas	1 h	1.65 ± 0.14	1.30 ± 0.18	2.94 ± 0.43	1.81 ± 0.22	1.05 ± 0.12	1.19 ± 0.15
	2 h	0.82 ± 0.10	0.75 ± 0.11	1.18 ± 0.31	0.93 ± 0.15	0.88 ± 0.11	0.71 ± 0.10
Liver	1 h	1.02 ± 0.16	2.20 ± 0.33	2.54 ± 0.46	2.28 ± 0.31	1.40 ± 0.26	2.31 ± 0.38
	2 h	0.91 ± 0.10	1.83 ± 0.24	2.0 ± 0.37	1.71 ± 0.20	1.12 ± 0.19	1.50 ± 0.27
Intestines^a^	1 h	0.86 ± 0.12	0.89 ± 0.12	0.95 ± 0.27	1.01 ± 0.17	0.90 ± 0.12	1.05 ± 0.18
	2 h	0.61 ± 0.06	0.50 ± 0.10	0.54 ± 0.16	0.76 ± 0.12	0.60 ± 0.10	0.73 ± 0.13
Muscle	1 h	0.23 ± 0.03	0.28 ± 0.04	0.32 ± 0.06	0.36 ± 0.07	0.30 ± 0.06	0.39 ± 0.03
	2 h	0.19 ± 0.02	0.20 ± 0.02	0.26 ± 0.05	0.28 ± 0.04	0.21 ± 0.03	0.23 ± 0.02
Kidneys	1 h	4.93 ± 0.38	4.85 ± 0.43	6.03 ± 1.0	6.52 ± 0.60	5.94 ± 0.63	5.12 ± 0.69
	2 h	4.01 ± 0.41	3.74 ± 0.24	5.0 ± 0.77	3.96 ± 0.48	3.20 ± 0.32	3.56 ± 0.50
Urine + bladder	1 h	64.0 ± 9.15	66.0 ± 6.60	61.0 ± 9.93	58.0 ± 6.25	62.0 ± 6.28	54.0 ± 5.78
	2 h	70.0 ± 8.70	68.0 ± 7.55	78.0±5.89	65.0 ± 9.13	71.0 ± 9.68	61.0 ± 7.10
Tumor	1 h	2.32 ± 0.25	2.09 ± 0.33	1.84 ± 0.40	2.15 ± 0.33	1.82 ± 0.26	1.78 ± 0.40
	2 h	1.65 ± 0.19	1.62 ± 0.18	1.30 ± 0.37	1.37 ± 0.25	0.84 ± 0.13	1.25 ± 0.21

^a^Parts of the intestines were measured without their contents

**Fig. 6:**
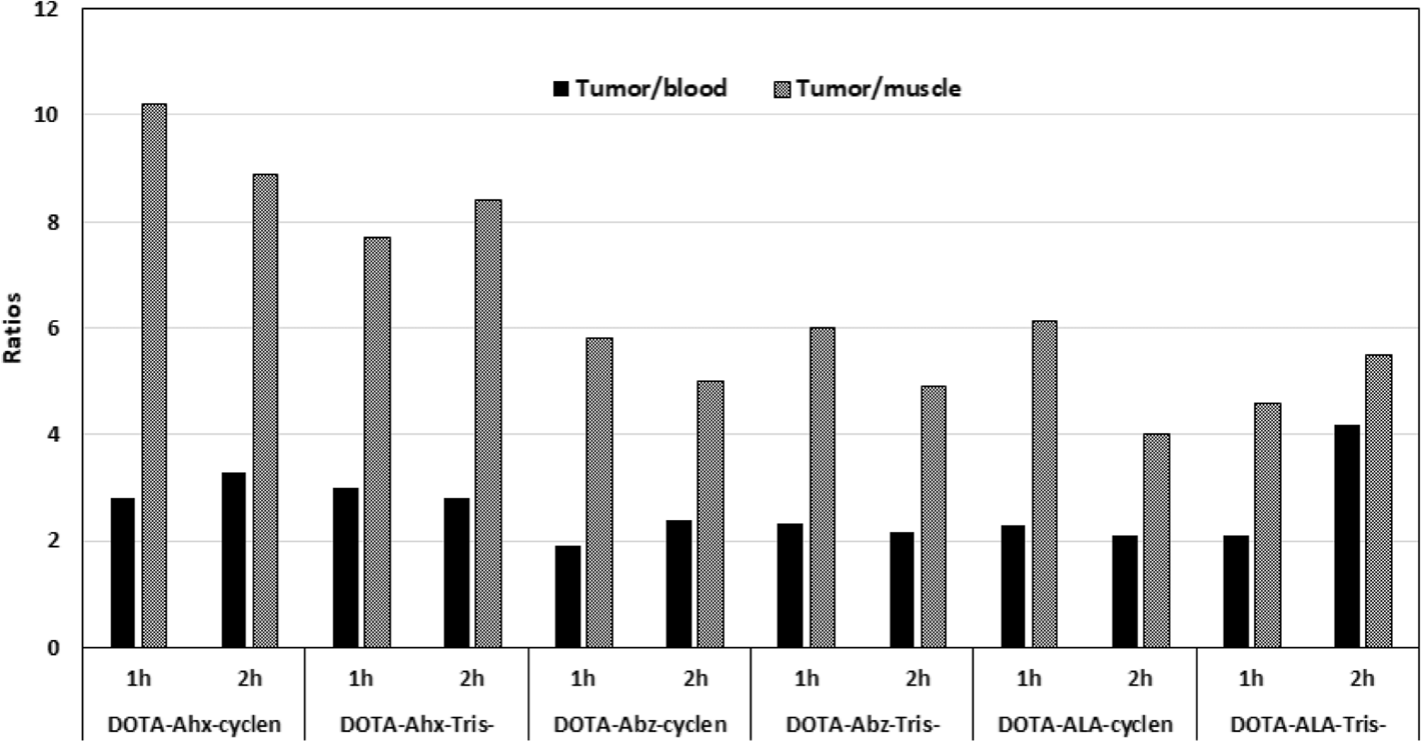
Tumor-to-blood and tumor-to-muscle uptake ratios for the ^68^Ga-DOTA-BN peptides in MDA-MB-231 xenografted nude mice at 1 and 2 h post-injection

Although the uptake in tumors was not very high for any of the BN peptides evaluated (up to 2.32 ± 0.25% ID/g), a somewhat decent tumor retention with slow washout from the tumors was observed for some of these peptides and especially for ^68^Ga-DOTA-Ahx-BN (28% washout over 2 h). Nonetheless, the uptake values in the tumors was always higher than the radioactivity found in the blood and muscle for all the radiopeptides. Tumor-to-nontumor uptake ratios were also calculated and graphically present in Fig. 6. A mixed trend of increased tumor-to-blood and tumor-to-muscle uptake ratios over time was observed for all the ^68^Ga-labeled BN peptide analogs. The tumor-to-blood uptake ratios ranged between 1.9 to 3.0 at 1 h, 2.0 to 4.2 at 2 h p.i., whereas tumor-to-muscle uptake ratios ranged between 4.6 to 10.3 at 1 h and 4.0 to 8.8 at 2 h p.i. A low bone uptake was detected for all the radioconjugates indicating high in vivo stability of these ^68^Ga-complexes. Similar to normal mice, the main route of excretion in tumor-bearing mice was through the kidneys into the urine, with the radioactivity excreted into the urine was up to 78% ID, whereas the hepatobiliary excretion (liver + intestines) of these radiopeptides were below 12% ID. A fairly good uptake and retention by the tumors combined with good tumor to background uptake ratios advocating the possible potential of these BN peptides in general, and BN peptide with Ahx spacer in particular, for targeting breast cancer in vivo. Again, the data from this set of experiments further confirm that the method of DOTA synthesis (cyclen or tris) has minimal effect on the biological and tumor-targeting properties of the BN peptides under investigation as both exhibited equally good tumor-targeting potential.

It is worth emphasizing that the scope of this article was not to compare the newly synthesized BN peptides with the published BN peptides but to compare the two different synthetic approaches (DOTA-tris and cyclen) for the preparation of DOTA-BN peptides. Since the sequences of the BN analogs used in this study are new in terms of the presence of some specific amino acids, therefore, a direct comparison with the previously published BN may be misleading. On the other hand, most of the DOTA-coupled BN (7–14) normal sequence (Gln^7^-Trp^8^-Ala^9^-Val^10^-Gly^11^-His^12^-Leu^13^-Met^14^-CONH_2_) exhibited the tumor uptake value in PC-3 tumor xenografts in the range of 2.5 to 4% ID/g [14, 15], which is somewhat similar to the values obtained with the BN peptides prepared and evaluated in this study. This indicates that that the different synthetic approaches used here has minimal effect on the tumor-binding characteristics of the resultant BN peptide analogs.

#### PET imaging of ^68^Ga-DOTA-Ahx-BN in MDA-MB-231 tumor-bearing nude mice models

**Fig. 7:**
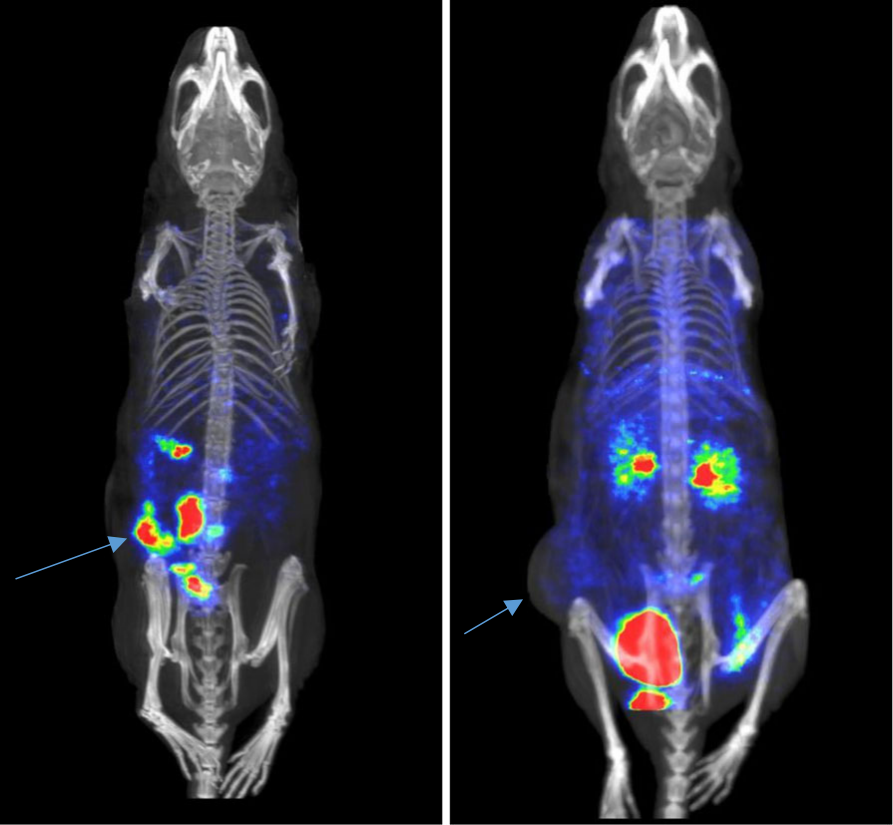
Whole body PET images in representative nude mice with MDA-MB-231 tumor from three groups tested. Coronal PET images obtained after 60-min post-injection of ^68^Ga-DOTA-Ahx-BN peptide (~ 100 ng, ~ 100 μCi). (Left) Tumor is clearly visible in the image suggesting receptor-specific tumor uptake. (Right) Tumor is hardly detectable in PET image under blocking condition in the presence of 300 μg cold BN peptide to block BN/GRP receptors. Arrows indicate the location of tumors

PET images of BN/GRP receptor-positive MDA-MB-231 tumor-bearing nude mice injected with ^68^Ga-DOTA-Ahx-BN peptide alone or along with a large excess of unlabeled BN peptide to block BN/GRP receptor sites obtained at 1 h p.i. are shown in Fig. 7.

Accumulation of radioactivity in the tumor lesion is clearly visible in PET image of the unblocked MDA-MB-231 tumor-bearing mouse at 1 h p.i., underlining the tumor-targeting ability of this radiotracer. Nonetheless, the radiopeptide showed considerable accumulation in the abdominal region including stomach and intestines. High amount of radioactivity was also seen in the urinary bladder, suggesting that the radiopeptide excreted mainly by the renal system.

Blocking studies in the presence of 200 μg of unlabeled BN peptide was also performed to demonstrate BN/GRP receptor-specific tumor uptake of ^68^Ga-DOTA-BN peptide. Unlike in the unblocked mouse PET imaging, where the tumor was clearly seen in the image, tumor was hardly detectable in the PET image under blocking conditions as shown in the Fig. 7. It was found that the presence of a pharmacological dose of BN peptide greatly reduced the tumor uptake, demonstrating the tumor specificity of ^68^Ga-DOTA-BN peptide. A low uptake was observed in the abdominal region but the radioactivity accumulation in the kidneys was higher than the unblocked mouse (Fig. 7). Intense radioactivity was also noted in the urinary bladder as the radiopeptide excreted primarily through the kidneys into the urinary bladder.

Immediately after imaging, the mice were sacrificed and tissue biodistribution were carried out in order to confirm the findings of PET-imaging. The data from the in vivo imaging was in agreement with the in vivo quantitative biodistribution studies (Table 4).

Based on encouraging biological and tumor-targeting properties, ^68^Ga-DOTA(cyclen)-Ahx-BN was further tested in nude mice bearing PC3 prostate carcinoma xenografts. Again in PC3 animal models, ^68^Ga-DOTA(cyclen)-Ahx-BN displayed fast and efficient clearance from the blood and other major organs. Kidney was the only major organ which retained most of the radioactivity (up to 6% ID/g) after 2 h p.i. The uptake of this radiotracer in the PC3 tumors was found to be 2.05 ± 0.54% ID/g at 1 h and 1.34 ± 0.22% ID/g at 2 h p.i., respectively. The tumor-to-blood and tumor-to-muscle ratios were 3.27 and 12.62 at 1 h and 2.85 and 11.40 at 2 h p.i., respectively (data not shown). A fairly comparable tumor uptake and pharmacokinetic profile was obtained between the nude mice models carrying estrogen receptor-independent MDA-MB-231 breast xenografts and androgen receptor-independent PC3 prostate cancer xenografts.

It is obvious from the biological evaluation that the ^68^Ga-DOTA(cyclen)-Ahx-BN peptide displayed equally good tumor-targeting properties in the species carrying two pharmacologically distinct human breast (MDA-MB-231) and prostate cancer cells (PC3), highlighting the ability of this radiotracer to target breast as well as prostate cancer in vivo. Taken together, the DOTA-coupled BN peptides prepared either from cyclen or tris displayed nearly similar in vivo tumor-targeting behavior (see Table 4), indicating equal tumor-targeting efficiency and potency for BN/GRP receptor positive tumors.

## Conclusions

In summary, an efficient and cost-effective synthetic procedure where a DOTA-linked peptide amide is synthesized on a single solid-phase support is presented in this study. One distinct feature of this approach is that the DOTA is introduced to the peptide backbone while the peptide still attached to resin support using standard SPPS methodology. Our results demonstrate that DOTA prepared from cyclen on solid-phase support showed comparable potency and efficiency to commercially available DOTA-tris in both in vitro and in vivo evaluation. The attractive synthetic methodology described here allows versatile, site-specific introduction of DOTA into peptides and can be applied to the synthesis of a wide variety of DOTA-linked tumor-targeting peptides to facilitate the development of molecular imaging and therapy (upon labeling with ^177^Lu) agents with optimal tumor-targeting properties for potential clinical translation.

## Data Availability

All data generated or analyzed during this study are included in this manuscript and its supplementary information files (mass spectrometric analysis and HPLC chromatograms) are also available from the corresponding author.

## Abbreviations

^68^Ga: Gallium-68
Abz: Aminobenzoic acid
Ahx: Aminohexanoic acid
ALA: Aminolevulinic acid
B_max_: Maximum binding sites/cell
BN: Bombesin
CH_3_CN: Acetonitrile
CT: Computed tomography
cyclen: 1,4,7,10-tetraazacyclododecane
DCM: Dichloromethane
DIC: Diisopropylcarbodiimide
DIEA: *N*,*N*-Diisopropylethylamine
DMF: *N*,*N*′-Dimethylformamide
DO3A: 1,4,7,10-Tetraazacyclododecane-1,4,7-triacetic acid
DOTA: 1,4,7,10-tetraazacyclododecane-*N*,*N*′,*N*′′,*N*′′′-tetraacetic acid
EtOH: Ethanol
FBS: Fetal bovine serum
Fmoc: 9-Fluorenylmethoxycarbonyl
GRP: Gastrin-releasing peptide
HATU: 1-[Bis(dimethylamino)methylene]-1*H*-1,2,3-triazolo[4,5-*b*]pyridinium 3-oxid hexafluorophosphate, *N*-[(Dimethylamino)-1*H*-1,2,3-triazolo-[4,5-*b*]pyridin-1-ylmethylene]-*N*-methylmethanaminium hexafluorophosphate *N*-oxide
HBTU: *N*,*N*,*N*′,*N*′-Tetramethyl-*O-*(1*H*-benzotriazol-1-yl)uronium hexafluorophosphate, *O*-(Benzotriazol-1-yl)-*N,N,N′,N′*-tetramethyluronium hexafluorophosphate
HOBt: Hydroxybenzotriazole
ID: Injected dose
*K*_d_: Dissociation constant
MBHA: 4-Methylbenzhydrylamine
MeOH: Methanol
MRI: Magnetic resonance imaging
MS: Mass spectrometry
p.i.: Post-injection
PBS: Phosphate-buffered saline
PET: Positron-emission tomography
ROI: Region of interest
RP-HPLC: Reversed-phase high performance liquid chromatography
SD: Standard deviation
SPECT: Single-photon emission computed tomography
SPPS: Solid-phase peptide synthesis
TFA: Trifluoroacetic acid
TIS: Triisopropylsilane
